# Physical Activity and Transitioning to Retirement: A Systematic Review

**DOI:** 10.1016/j.amepre.2012.05.026

**Published:** 2012-09

**Authors:** Inka Barnett, Esther M.F. van Sluijs, David Ogilvie

**Affiliations:** MRC Epidemiology Unit and UK Clinical Research Collaboration Centre for Diet and Activity Research (CEDAR), Cambridge, United Kingdom

## Abstract

**Context:**

The transition to retirement has been recognized as a turning point in determining physical activity and may present a critical “window” for promoting physical activity. This systematic review examined changes in physical activity across the retirement transition, whether these changes vary by SES, and what is known about predictors of these changes.

**Evidence acquisition:**

Peer-reviewed articles and gray research literature, published between January 1980 and July 2010 in any country or language, were identified. Longitudinal and cross-sectional observational studies were included. Study selection, quality assessment, data extraction, and synthesis were performed between July 2010 and March 2011. A harvest plot approach to visualizing the findings was combined with a narrative synthesis.

**Evidence synthesis:**

Of the 19 included studies, 11 examined changes in exercise, or leisure-time physical activity, or both; seven, changes in total physical activity; and one study, both. Most studies used single-item measures of physical activity (*n*=9) or custom questionnaires (*n*=6). Results suggested that exercise and leisure-time physical activity increased after the retirement transition, whereas findings regarding total physical activity were inconsistent. SES moderated the association, with low SES being associated with a decrease and high SES with an increase in physical activity. Evidence on predictors of change was scarce and methodologically weak.

**Conclusions:**

Evidence suggests that exercise and leisure-time physical activity increases after the retirement transition, but whether and how total physical activity changes is unclear. Imprecise physical activity measures used in primary studies limit conclusions, and this highlights the need for further research.

## Context

The global population is aging rapidly. It is estimated that 21% of the population will be aged >60 years by 2050.^1^ A major challenge of the growing number of old people is the potential increase in healthcare demands due to age-related chronic diseases and disabilities.^2^ Physical activity has been shown to be crucial for reducing the risk of cardiovascular diseases, diabetes, and some cancers; prevention of falls; and maintenance of independence in the later years of life.^3–8^ Despite these benefits, objectively measured physical activity levels are consistently low in older adults.^9–11^

Retirement has been recognized as a critical turning point in determining physical activity behaviors in old age. Retirement may therefore present an important ‘window’ for targeting interventions to promote increases in, or maintenance of, overall physical activity levels.^12–14^ Retirement is a major life transition that is associated with life changes including those involving social networks, income, and time flexibility that may all affect physical activity behaviors.^15–17^ As governments around the world discuss reforms of existing retirement schemes to account for the rising number of retirees, it is important to gain better insight into the effects of retirement on protective health behaviors such as physical activity.

In recent decades, the concept of retirement has become increasingly multifaceted and there is no consensus among researchers on how to define retirement satisfactorily.^17^ Rather than a single event of permanent withdrawal from working life, retirement has developed into an individualistic and sometimes prolonged transition process. Emerging new forms of and pathways to retirement such as bridge employment and gradual or partial retirement add further complexity.^17–19^ This paper focuses on the transition to old-age retirement, that is, retirement at around the official retirement age of about 65 years in most countries.^2^ To reflect the evolving conceptualizations of retirement and account for actual retirement patterns in various countries, as opposed to those implied by regulations regarding retirement, an exploratory approach was adopted, rather than a restrictive definition, to allow for differing understandings of and approaches to old-age retirement.

Socioeconomic status is associated with physical activity in adults, with people from lower SES strata being consistently less physically active.^20,21^ However, whether SES influences changes in physical activity across the transition to retirement has not been reviewed systematically. Moreover, a better understanding of the predictors of change in physical activity across the retirement transition is important in order to design future interventions effectively. To our knowledge, no review of evidence on physical activity and the transition to retirement has been conducted to date. Therefore, the aims of this systematic review are to investigate (1) changes in physical activity across the transition to retirement; (2) whether these changes vary by SES; and (3) what is known about predictors of changes in physical activity across the retirement transition.

## Evidence Acquisition

### Search and Selection

Based on the findings of an initial scoping of the literature to assess the feasibility of a full systematic review, a literature search was performed using a range of electronic databases: Abstracts of Social Gerontology, AMED, Anthropology Plus, ASSIA, BNI, CINAHL, Cochrane Library, EMBASE, ISI Web of Science, JSTOR, MEDLINE, Physical Education Index, ProQuest Digital Dissertations, PsycINFO, Science Direct, Scopus, Social Service Abstracts, Sociological Abstracts and SportDiscus. To minimize publication bias and to fully reflect the available evidence, both peer-reviewed journal articles and gray research literature including published PhD dissertations, research reports, and full conference papers were considered. Evidence published between January 1980 and July 2010 in any country or language was eligible. Search terms used referred to retirement (exposure) and physical activity (outcome; see Table 1 for the search syntax as applied in MEDLINE). A hand-search of five peer-reviewed electronic journals specializing in aging—*Age and Ageing*, *Age & Society*, *European Review of Aging and Physical Activity*, *Journal of Aging and Health*, and the *International Journal of Aging & Human Development*—and a search of the citations and reference lists of the included studies complemented the search.

### Inclusion and Exclusion Criteria

Eligible studies examined free-living physical activity (as opposed to that in controlled laboratory conditions) and had longitudinal or cross-sectional observational designs. Longitudinal studies had to measure physical activity across the transition to retirement within the same individual. Cross-sectional studies had to compare physical activity in retired and nonretired individuals. Only quantitative studies were considered.

Studies that assessed physical activity as a matter of necessity (occupational, transport, and household activities) as well as for recreational purposes were included. Studies were excluded if they (1) examined only physical capability or physical fitness; (2) investigated only pre- or post-retirement physical activity without referring to the transition or the difference; (3) examined physical activity only as a side exposure or covariate and not as the main outcome in analysis; (4) assessed retirement and physical activity concurrently, but only as these two related to a third variable or condition; (5) assessed preference for physical activity and not actual behavior; or (6) examined sedentary behaviors only. Studies that investigated solely temporary or early (aged <50 years) retirees or retired professional athletes were not included, as participants were likely to be substantially younger or to suffer from health problems that could affect physical activity behavior.

### Screening

Titles and abstracts of all citations initially were screened against the inclusion and exclusion criteria by one reviewer, and a random sample of 10% was cross-checked by a second reviewer. Retained full-text articles were screened independently by the two reviewers against the criteria. Any disagreements were resolved by discussion. Studies that were excluded in the full-text screening were recorded with reasons for exclusion.

### Data Extraction and Quality Assessment

A data extraction form was developed and piloted with two studies. The following data were extracted by one reviewer: authors, study setting, sample characteristics, objectives, methodology, outcome and exposure measures, results and predictors of physical activity across the transition to retirement. A second reviewer cross-checked the data extracted from all included studies. Disagreements were resolved through discussion.

The study validity was assessed by two reviewers independently using the Critical Appraisal Skills Programme (CASP; www.casp-uk.net) checklist for cohort studies. The checklist consists of 12 questions that were judged suitable for assessing both the longitudinal and cross-sectional studies as they evaluate internal validity (based on potential measurement biases and confounding) and external validity (based on potential selection bias). After initial assessment of all studies based on the 12 questions, the questions were collapsed into four criteria that were used to summarize the overall quality of each study and rank the studies (Table 2). Minimum thresholds were defined for each criterion, and studies that met the threshold received a point. The sum of points awarded represented the overall quality score of a study. A study was considered of low quality if the score was 0–1, modest quality if the score was 2, and high quality if the score was 3–4 (Appendix A, available online at www.ajpmonline.org).

### Data Analysis

As the outcome measures of the primary studies were very heterogeneous, meta-analysis was considered inappropriate. A harvest plot approach developed by Ogilvie and colleagues^41^ was used for visualizing the findings. The approach emulates the graphical presentation of a forest plot by enabling findings to be visualized in a way that takes study quality into account. A hypothesis-testing approach was adopted, comparing the null hypothesis of no change in physical activity across the transition to retirement with two alternative hypotheses of a positive and a negative change, respectively.

Each plot consisted of three columns representing the three competing hypotheses, and each study was represented by a bar in the column for the hypothesis with which its results were most consistent. The rows represented the domains of physical activity assessed in the studies: exercise, leisure-time, and total physical activity. In these studies, exercise included planned and structured physical activities such as sports, jogging, or swimming; leisure-time physical activity included exercise but also hobbies and other active recreation; and total physical activity included physical activities in at least two domains of daily living (leisure-time or exercise, occupational, transport or household).

The height of the bars indicated the quality of each study based on the scoring. The shading of the bars illustrated the sample size. The harvest plots were combined with a narrative synthesis. Study selection, quality assessment, data extraction, and synthesis were performed between July 2010 and March 2011.

## Evidence Synthesis

**Figure 1:**
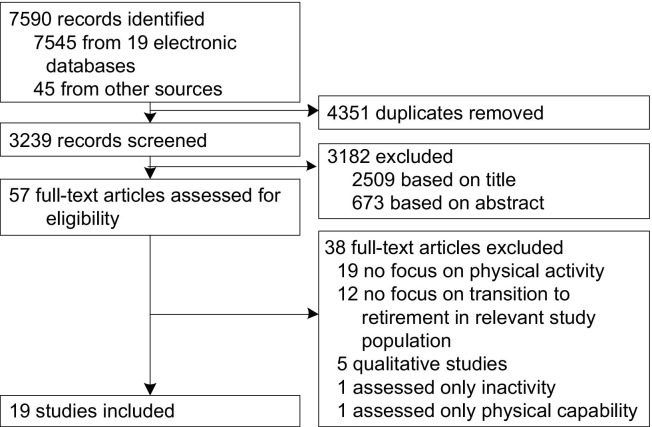
Review flowchart of search results

The initial search yielded 7635 citations. After removing duplicates, 3239 publications remained. Following screening of the titles and abstracts, 57 full-text papers in four languages were obtained for in-depth review. Of those, 38 were excluded, the majority because they did not examine change in physical activity or the transition to retirement. Of the remaining studies, 19 were included in the current review (Figure 1).

### Study Characteristics and Methodologic Quality

Fifteen journal articles^22–36^ and four published PhD theses^37–40^ were included. The characteristics of the studies reviewed are summarized in Table 3; for a detailed description of the included studies, see Appendix B (available online at www.ajpmonline.org). Most studies were found to be of modest (*n*=9) or low (*n*=7) methodologic quality.

Thirteen studies were published after 2000 and ten were set in the U.S. The majority of studies (*n*=14) were prospective cohort studies with follow-up periods of between 2^30^ and 13 years^33^ (M=4.8, SD=3.0) and five studies^26,29,31,35,36^ were cross-sectional. Four studies^24,37,38,40^ presented results from analyses of different waves of the same cohort (the Health and Retirement Survey). Relevant studies focused on changes in exercise, leisure-time, or total physical activity. Two studies^22,33^ reported findings on two domains of physical activity.

Almost all physical activity data were ascertained using self-report questionnaires, with considerable reliance on single-item questions^24,26,30,32,35–38,40^ (*n*=9) and custom questionnaires^22,27,29,31,33,39^ (*n*=6) of unknown validity. Only three studies^23,25,34^ used validated physical activity questionnaires. One study^32^ reported objective measures of physical activity; however, the outdated measurement technology (under-heel pressure transducers for measuring footfall) and other methodologic limitations (such as considerable selection bias and lack of adjustment for confounders) of this small-scale study from 1986 limit the transferability of the results.

### Association Between Retirement and Physical Activity

**Figure 2:**
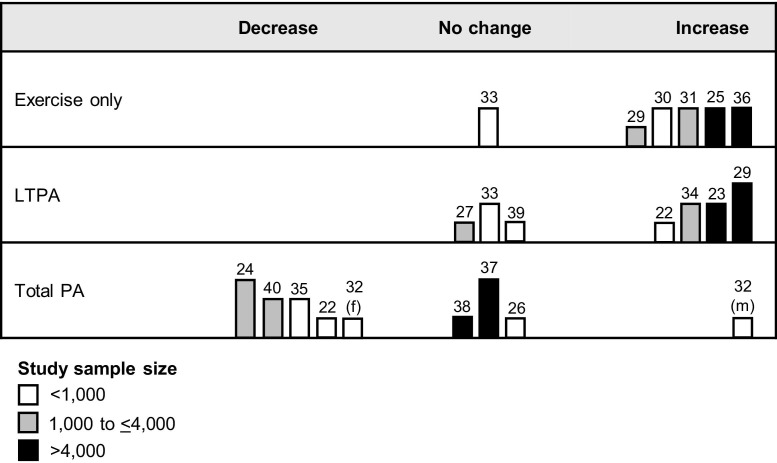
Harvest plots of evidence for changes in physical activity across the transition to retirement *Note:* Study quality scores are indicated by the height of the bar (short=0 or 1; medium=2; tall=3 or 4). Numbers above the bars refer to study citations. f, female; m, male

Figure 2 shows the harvest plots for physical activity changes across the transition to retirement. Studies were categorized according to the physical activity domains examined, the domains being presented in order from the narrowest to the broadest definitions of physical activity. Overall, exercise and leisure-time physical activity increased after the transition to retirement, whereas the findings regarding changes in total physical activity were inconclusive.

Five studies examined changes in exercise.^25,28,30,31,36^ Most of these studies met two of the four quality criteria. The heterogeneity of the measures of exercise prevented an estimation of an overall magnitude of the increase in exercise. However, significant results from a large longitudinal study^25^ that employed a validated questionnaire suggest that inactive participants were more likely to adopt exercise after the transition to retirement than participants who remained employed. In a large cross-sectional study,^36^ retired individuals were almost twice as likely as those still working to engage in regular exercise (*p*<0.001).

Six studies examined changes in leisure-time physical activity.^22,23,27,29,34,39^ Most studies met one or two of the four quality criteria; one study^29^ met three criteria. Significant results from a longitudinal study^34^ that employed validated questionnaires included an estimate of an increase in leisure-time physical activity of 2.1 hours per week in men and 1.8 hours per week in women. Another large longitudinal study estimated that retired women had 1.54 times higher odds (95% CI=1.24, 1.91, *p*<0.0001) of an increase in leisure-time physical activity compared to women who were still working.^23^

One study^33^ examined changes in both exercise and leisure-time physical activity. This study received a modest quality rating. In this study, no evidence for an increase or decrease in either exercise or leisure-time physical activity after the transition to retirement was found.

Eight studies^22,24,26,32,35,37,38,40^ examined changes in total physical activity. Methodologic quality varied. Most studies used single questions to assess changes in total physical activity. Findings were inconsistent. Five studies^22,26,35,37,40^ (including one high-quality study^37^) reported a decrease in total physical activity, and three studies^24,26,38^ (including one high-quality large study^24^) reported no change. Of the studies that did not find an association, two^26,38^ had weak study designs, including one small cross-sectional study.^26^ The third study^24^ found no consistent change in total physical activity among all study participants but reported a decrease of 7.5% in people who retired from physically demanding occupations, whereas total physical activity increased by 4.4% in retirees from sedentary jobs. One study^32^ reported an increase in total physical activity in men and a decrease in women, but this study was limited by outdated measurement techniques and other methodologic weaknesses, as discussed above.

### Socioeconomic Patterns in the Impact of Retirement on Physical Activity

Four studies^22,24,29,37^ examined whether physical activity change in retirement differed by SES. All studies used occupation to define SES, and two studies^24,37^ used household wealth as an additional indicator. Retirement from manual or low-grade occupations was consistently associated with lower physical activity after retirement. Three studies^22,24,29^ reported a positive association between physical activity and retirement from sedentary or high-grade occupations, whereas one study^37^ found a nonsignificant decline in previous sedentary workers and a significant decline in previous manual workers.

When wealth was introduced as an additional factor, one study^37^ found a decrease in total physical activity in people from both higher- and lower-wealth groups, but the decline was almost double in retirees with lower wealth. Chung et al.^24^ identified an interaction effect between wealth and occupational class. Lower wealth increased the negative association between retirement from a manual occupation and physical activity. Higher wealth increased the positive association between retirement from a sedentary occupation and physical activity.

### Predictors of Change in Physical Activity

Table 4 summarizes potential predictors of change in physical activity across the transition to retirement that were examined in the included studies. The most consistent evidence identified was related to gender. Although the direction of change in physical activity was consistent for both genders in four^25,29,30,34^ of five studies, the increase in physical activity was slightly higher in men than women. Evidence on other predictors was scarce, often inconsistent, and methodologically weak.

## Discussion

The main findings of this systematic review were that exercise and leisure-time physical activity increased with the transition to retirement, whereas no clear pattern emerged with regard to total physical activity. SES seemed to moderate the association, with lower SES consistently being associated with a decrease in physical activity and higher SES with an increase.

A postretirement increase in exercise and leisure-time physical activity is not unexpected, as at least some of the additional time available after exit from the labor force is likely to be used for physical activities. Further research, including qualitative studies, is needed to better understand the underlying motives for an increase in exercise and leisure-time physical activity after the transition to retirement and how these can be used to promote adoption of physical activity among recent retirees. Longitudinal studies with longer follow-up are needed to examine whether the increase in exercise and leisure-time physical activity is maintained in the long term or is only a short-term phenomenon after the transition to retirement.

The inconclusive findings regarding total physical activity may be explained by the various assessment tools used and their abilities to capture all physical activities within the different domains (occupation, transportation, domestic and leisure) that contribute to total physical activity. There is a need for more research with validated domain-specific physical activity questionnaires, objective measures, or both to gain a fuller understanding of changes in total physical activity.

The results on the moderating effect of SES on physical activity change in retirement are in agreement with findings from a previous systematic review^21^ that found consistently lower levels of physical activity in middle-aged adults from lower SES strata.^21^ No pathways that could explain these associations were proposed in the included studies; however, lack of financial resources in retirement, preference for sedentary activities, negative experiences of participation in physical activity when younger, unsafe neighborhood environments, and lower awareness of the benefits of physical activity have been proposed as key barriers specific to physical activity among retirees from low-SES backgrounds.^42^ The findings on SES need to be interpreted with caution because robust assessment of SES can be challenging in retirement.^43^ For example, the use of the last occupation to determine SES might lead to misclassification because of the downward mobility experienced by many employees in later working life. Further studies with other measurements of SES are needed to confirm the association.

The review also highlights the need for more research on predictors of physical activity change across the transition to retirement. There was some consistent evidence suggesting that men increase their physical activity more than women after the transition to retirement. However, the robustness of this conclusion and its public health implications require further investigation.

### Methodologic Issues

A key methodologic limitation of many of the primary studies was the imprecise and self-reported nature of the physical activity measures used. The shortcomings of self-reported physical activity assessments have been discussed widely and summarized in the literature. They include limitations in reliability, validity and sensitivity to change, challenges in assessing the frequency, duration and intensity of activities, and the risk of social desirability and recall bias.^44–46^

Most of the primary studies (*n*=9) employed single-item physical activity assessments that are unlikely to capture all the physical activities of daily living sufficiently and might have low responsiveness to change in physical activity.^47–49^ In four studies,^24,37,38,40^ participants were asked about total vigorous physical activity only (defined as sports, heavy housework, or a job that involves physical labor) which might have led to underestimation, as elderly individuals have been shown to prefer light- to moderate-intensity physical activity because of physical limitations.^50^ Only one study^32^ used objective physical activity measures; however, the multiple other shortcomings of this study limit the transferability of its findings. The limitations in the assessment of physical activity highlight the need for more research with age-appropriate, validated, and domain-specific physical activity questionnaires, or objective measures, or both to gain a fuller understanding of changes in physical activity across the transition to retirement.

Almost all primary studies (*n*=15) defined retirement as a permanent and complete withdrawal from the labor force. Of the four remaining studies, one study^24^ included as “retired” those participants who were not permanently retired, and three studies^25,29,35^ included those who were still working after retirement from their main occupation (e.g., voluntary work, self-employment or part-time work). Length of and reasons for retirement were considered in only two studies.^28,35^ The time since retirement might make a difference to individuals' adjustments to and views on retirement^51,52^ and consequent lifestyle behaviors.

Similarly, the reasons for retirement might affect motivation, financial means, and physical ability to be physically active. For example, people who retired because of physical or mental health problems or to care for an ill spouse or relative might lack motivation, time, and capability to be physically active after retirement. In contrast, those retiring early by choice might be highly motivated and have access to sufficient financial resources—thanks to generous early retirement packages—to increase their physical activity levels. Future studies should take the potential complexity of the exposure of “retirement” into account**.**

This review synthesized studies from seven different countries, among which comparability might be limited. Various social welfare and public pension systems might affect retirement decisions^53^; retirement income^54,55^; and (ultimately) retirement behaviors including physical activity. This should be considered in more detail in future work.

### Strengths and Limitations

A key strength of this paper is that it is the first systematic review of the literature on changes in physical activity across the transition to retirement. This review did not apply any language or country restrictions and included published peer-reviewed journal articles as well as gray research literature, minimizing the chance of publication and language bias. The search of a large number of databases plus reference lists and hand-searching reduced the risk of selection bias.

Literature databases from multiple disciplines were used and the search strategy was purposefully broad to capture literature from a wide range of academic fields. Consequently, the present review included studies carried out by economists and social scientists as well as health researchers. Although providing a more comprehensive picture of the body of literature available, this multidisciplinary approach also contributed to the heterogeneity of the results and therefore, perhaps, to the unfeasibility of meta-analysis.

### Conclusion

This systematic review has shown that exercise and leisure-time physical activity increase after the transition to retirement, but whether and how total physical activity changes remains unclear and needs further research. People who retire from low-grade or manual occupations appear to be particularly vulnerable to low levels of physical activity in retirement. However, further studies with other measures of SES are needed to confirm this association. Additionally, further research should include appropriate and valid physical activity measures, apply clear and relevant definitions of retirement, and study predictors of physical activity change across the transition to retirement.

## Figures and Tables

**Table 1:** Search terms used for MEDLINE search

Concept	Search terms
Retirement	(retirement [MESH Terms] OR retir*[Title/Abstract] OR pensio*[Title/Abstract] OR “late life transitio*”[Title/Abstract])
Physical activity	(physical* activ*[Title/Abstract] OR Motor Activity[MeSH Terms] OR Exercise[MeSH Terms] OR Sports[MeSH Terms] OR recreation[MeSH Terms]OR leisure activ*[Title/Abstract]

**Table 2:** Quality assessment of the included studies

Author	Selection bias	Measurement bias		Confounding	Overall (0–4)^a^
		Retirement	Physical activity		
Berger (2005)^22^	No	Yes	Yes	?	1
Brown (2009)^23^	Yes	No	No	Yes	2
Chung (2009)^24^	No	No	Yes	No	3
Evenson (2002)^25^	Yes	No	No	Yes	2
Fonseca (2004)^26^	Yes	No	Yes	Yes	1
Glamser (1985)^27^	Yes	No	Yes	Yes	1
Henkens (2008)^28^	Yes	No	Yes	?	1
Mein (2005)^29^	No	No	Yes	No	3
Midanik (1995)^30^	No	No	Yes	Yes	2
Parnes (1985)^31^	Yes	No	Yes	No	2
Patrick (1985)^32^	Yes	?	No	Yes	1
Slingerland (2007)^33^	Yes	No	Yes	No	2
Touvier (2010)^34^	Yes	No	No	Yes	2
Wells (1999)^35^	No	No	Yes	Yes	2
Wister (1996)^36^	No	No	Yes	?	2
Chung (2005)^37^	No	No	Yes	No	3
Nekuda (2007)^38^	?	?	Yes	No	1
Parise (2006)^39^	?	Yes	Yes	Yes	0
Zheng (2008)^40^	?	No	Yes	No	2

*Note:* ?, not reported; studies ordered by type (published articles followed by PhD theses) and then alphabetically by first author

a 0–1 indicates low quality; 2 indicates modest quality; 3–4 indicates high quality.

**Table 3:** Characteristics of the included studies, *n* (%)

Characteristics	Value
**Study population**	
U.S.	10 (53)
United Kingdom	3 (16)
Australia	2 (11)
Netherlands	1 (5)
Portugal	1 (5)
Canada	1 (5)
France	1 (5)
**Type of sample**	
Population-based	14 (74)
Employee cohort	4 (21)
HMO insurance policy holders	1 (5)
**Gender of sample**	
Male	4 (21)
Female	2 (11)
Both genders	13 (68)
**Exposure measurement (retirement)**	
Self-reported retirement status	8 (42)
Self-reported employment status	9 (47)
Employer's records	1 (5)
As major life event	1 (5)
**Outcome measure (physical activity)**	
Self-reported, validated questionnaire	3 (16)
Self-reported, nonvalidated questionnaire	6 (32)
Based on single-item question	9 (47)
Objectively measured	1 (5)

*Note:* 19 studies were included.

**Table 4:** Summary of studies of predictors of physical activity change across the transition to retirement

Predictors	Association and studies		
	−	0	+
Gender (male)			25, 29, 30, 32, 34
Ethnic group (African- American vs white)	31	25	
Being married			31
Retirement from strenuous occupation	40	34	
Lifelong participation in physical activity		27, 39	38
Positive attitude toward retirement			31
Pathway to retirement (voluntary vs involuntary)		28	
Work after retirement from main occupation (part-time/full-time)	29		
Being retired for <5 years	35		

*Note:* Numerals represent reference numbers. Associations: −, negative; +, positive; 0, no association
