# Supplementary material for: Physical Activity and Transitioning to Retirement: A Systematic Review
Source: Am J Prev Med. 2012 Sep;43(3):329–36. doi: 10.1016/j.amepre.2012.05.026 (PMC3830178; doi:10.1016/j.amepre.2012.05.026)
Supplement: Appendix A and B [file mmc1.pdf]

Physical Activity and Transitioning to Retirement
A Systematic Review

Inka Barnett, MSc, Esther M.F. van Sluijs, PhD, David Ogilvie, PhD

Appendix A

Tool used for the quality appraisal of the primary studies, based on Critical Appraisal Skills Programme (CASP) checklist for cohort studies

12-question checklist	Criteria	Binary thresholds for criteria
1. Did the study address a clearly focused issue?<br>2. Did the authors use an appropriate method to answer their question?	Initial quality screening questions	
3. Was the cohort recruited in an acceptable way?<br>4. (a) Was the follow-up of the subjects complete enough?<br>(b) Was the follow-up of the subjects long enough?	Criterion 1: External validity assessment of selection bias	Random sample and nonresponse <30% and loss of follow-up <50%=Yes (1 point)<br>Nonrandom sample and/or nonresponse ≥30% and/or follow-up ≥50%=No (0 points)
5. Was the exposure (retirement) accurately measured to minimize bias?	Criterion 2: Internal validity assessment of measurement bias (exposure: retirement)	Self-reported retirement status or objective (e.g., based on company records, receipt of pension)=Yes (1 point)<br><br>Merged with participants that might not be retired (homemakers, unemployed, students)=No (0 points)
6. Was the outcome (physical activity) accurately measured to minimize bias?	Criterion 3: Internal validity assessment of measurement bias (outcome: physical activity)	Objective measurement (e.g., accelerometer) or validated questionnaire=Yes (1 point)<br>Custom questionnaire or single question=No (0 points)
7. (a) Have the authors identified all important confounding factors?<br>(b) Have they taken account of the confounding factors in the design and/or analysis?	Criterion 4: Internal validity assessment of confounding	Adjusted for all confounders (age, gender, SES, functional status) (analysis, stratification, or exclusion)=Yes (1 point)<br>Adjusted for some or none of the confounders=No (0 points)
8. What are the results of this study?<br>9. How precise are the results?<br>10. Do you believe the results?<br>11. Can the results be applied to the local population?<br>12. Do the results of this study fit with other available evidence?	Overall quality assessment of the results and transferability of the study findings (as determined by likelihood of selection bias (Criterion 1); measurement biases (Criteria 2 and 3); and confounding (Criterion 4). No additional quality criterion was used.	

Appendix B

Summary of the 19 studies on retirement and physical activity that were reviewed between July 2010 and August 2010

Study and country	Design (follow-up)	Participants (N, age, gender, retirement status)	Assessment of physical activity	Results	Quality ranking (0–4)
Berger (2005)[1] United Kingdom	Cohort (4.5 years) Population-based	N= 699<br>~60 years<br>45.5% male<br><br>At baseline: 49.5% employed, 50.5% not employed<br><br>At follow-up: 21.6% employed, 78.6% not employed	Leisure, home, and occupational physical activity questionnaire	Employed had lower odds of meeting physical activity recommendation with nonwork physical activity compared to non-employed (OR 0.59 [95% CI=0.4, 0.86])<br>Employed had higher odds of meeting physical activity recommendations with total physical activity compared to non-employed OR (2.72 [95% CI=1.88, 3.97])	Poor (1)
Brown (2009)[2] Australia	Cohort (3 years) Population-based	N= 8762<br>51–55 years<br>Women only<br>Not reported how many retired	Leisure physical activity<br>Active Australia physical activity survey[3]	Participants who retired were more likely to report an increase in leisure physical activity (compared to 3 years ago) than participants who were still employed (OR 1.54 [95% CI=1.24, 1.91])	Modest (2)
Chung (2009)[4] U.S.	Cohort (6 years) HRS (Wave 3-6) Population-based	N=11,469<br>Age not reported<br>47.1% male<br>30% retired at baseline<br>43% retired at follow-up<br>7% reversible retirement	Total vigorous physical activity<br>Single-item question on total vigorous physical activity	No significant change in total vigorous physical activity (three or more times a week) in retirement	Good (3)
Evenson (2002)[5] U.S.	Cohort (6 years) Population-based	N=7782<br>45–64 years<br>41.4% male<br>All employed at baseline<br>29.5% retired at follow-up	Leisure and occupational physical activity<br>Baecke questionnaire[6]	Exercise participation increased for all gender and ethnic groups in retirement<br>No results for occupational physical activity reported	Modest (2)
Fonseca (2004)[7] Portugal	Cross-sectional Population-based	N=100<br>M 59.4 years (SD 4.6)<br>50% male<br>50% retired	Total physical activity<br>Single-item question on total physical activity	No difference in total physical activity between retirees and employees	Poor (1)

Study and country	Design (follow-up)	Participants (N, age, gender, retirement status)	Assessment of physical activity	Results	Quality ranking (0–4)
Glamser (1985)[8]<br>U.S.	Cohort<br>(6 years)<br>Employee-based	N=51<br>~62 years<br>Male only<br>All employed at baseline<br>All retired at follow-up	Leisure physical activity<br>Based on list of leisure activities	No change in leisure-time physical activity in retirement	Poor (1)
Henkens (2008)[9]<br>Netherlands	Cohort<br>(6 years)<br>Employee-based	N=1604<br>M 54.8 years (SD 2.8)<br>75% male<br>All employed at baseline<br>55.1% retired at follow-up	Exercise physical activity<br>Single-item question on change in exercise	Participants who retired were more likely to report an increase in exercise (compared to 5 years ago) than participants who were still employed (RR 2.90 [95% CI=2.19, 3.84] in voluntary retired; RR 2.14 [95% CI=1.47, 3.13] in involuntarily retired)	Poor (1)
Mein (2005)[10]<br>United Kingdom	Cross-sectional<br>Employee-based	N= 6224<br>45–69 years<br>26.0% male<br>36.4% retired	Leisure and home physical activity<br>Questionnaire	Retirees were more likely to meet physical activity recommendations than participants who were still employed (OR 2.5 [95% CI= 1.9, 3.5] in women; OR 3.5 [95% CI=2.8, 4.3] in men)	Good (3)
Midanik (1995)[11]<br>U.S.	Cohort<br>(2 years)<br>HMO insurance holders	N=595<br>M 61.8 years (retired at follow-up)<br>M 62.3 years (employed at follow-up)<br>57.5% male<br>All employed at baseline<br>53.8% retired at follow-up	Exercise physical activity<br>Single-item question on exercise	Participants who retired were more likely to report participation in regular exercise than participants who were still employed (men: RR 3.5 [95% CI =2.0, 6.2]; women: RR 2.2 [95% CI=1.2, 4.0])	Modest (2)
Parnes (1985)[12]<br>U.S.	Cross-sectional<br>Population-based	N= 2899<br>57–71 years<br>Male only<br>60.6% retired	Exercise physical activity<br>Questionnaire	More retirees participated in exercise (in last 12 months) than employees (44% retired vs 35% employees, *p*<0.01)	Modest (2)
Patrick (1986)[13]<br>United Kingdom	Cohort<br>(2 years)<br>Employee-based	N=74<br>M 65.1 years (SD 0.2)<br>71.6% male<br>All employed at baseline<br>All retired at follow-up	Total physical activity<br>Objectively measured total physical activity plus physical activity diary	Total physical activity (time and intensity) increased in men after retirement and decreased in women	Poor (1)

Study and country	Design (follow-up)	Participants (N, age, gender, retirement status)	Assessment of physical activity	Results	Quality ranking (0–4)
Slingerland (2007)[14]<br>U.S.	Cohort<br>(13 years)<br>Population-based	N=971<br>M 50.5 years (SD 5.3)<br>75.0% male<br>All employed at baseline<br>70.4% retired at follow-up	Exercise, leisure-time, and work-related transport physical activity<br>Questionnaire	Participants who retired had lower odds of a decline in leisure-time physical activity compared to participants who were still employed (OR 0.36 [95% CI =0.19, 0.68])<br>No significant differences in changes in exercise physical activity in retired and employed<br>Participants who retired had higher odds of a decline in work-related transport physical activity compared to those who were still employed (OR 3.03 [95% CI =1.97, 4.65])	Modest (2)
Touvier (2010)[15]<br>France	Cohort<br>(3 years)<br>Population-based	N=1389<br>45–64 years<br>50.3% male<br>All employed at baseline<br>20.4% retired at follow-up	Leisure and occupational physical activity<br>Modifiable Activity Questionnaire (MAQ)[16]	Leisure physical activity increased in retirement (M 2.1 (SD 0.4) hours/week in men;<br>M 1.8 (SD 0.4) hours/week in women)<br>No results for occupational physical activity reported	Modest (2)
Wells (1999)[17]<br>Australia	Cross-sectional<br>Population-based	N=363<br>M 69.9 years (SD 4.0)<br>Female only<br>7.7% working<br>20.0% partly retired<br>27.0% recently retired<br>36.7 % long-term retired	Total physical activity<br>Single-item question on change in total physical activity	Retirees were more likely to report a decrease in total physical activity (compared to 5 years ago) than participants who were still employed (OR 3.89, $p<0.05$)	Modest (2)
Wister (1996)[18]<br>Canada	Cross-sectional<br>Population-based	N=5333<br>>45 years<br>44.5% male<br>18.0% retired	Exercise physical activity<br>Single-item question on exercise physical activity	Retirees were more likely to participate in exercise (once a week or more) than participants who were still employed (OR 1.73, $p<0.001$)	Modest (2)
Chung (2005)[19]<br>U.S.	Cohort<br>(2 years)<br>HRS (subsample 2001–2003)<br>Population-based	N=2762<br>M 63.5 years (SD 4.6)<br>40.7% male<br>All employed at baseline<br>55.7% retired at follow-up	Total vigorous physical activity<br>Single-item question on total vigorous physical activity	Total vigorous physical activity decreased in retirement (–3.5 [SD 1.05] MET hours/day, $p<0.01$)	Good (3)

Study and country	Design (follow-up)	Participants (N, age, gender, retirement status)	Assessment of physical activity	Results	Quality ranking (0–4)
Nekuda (2009)[20]<br>U.S.	Cohort<br>(2 years)<br>HRS (subsample 1998–2000)<br>Population-based	N= 5351<br>M 58.9 years (SD 6.7) remained employed<br>M 63.4 years (SD 6.9) retired<br>51.7% male<br>All employed at baseline<br>16.9% retired at follow-up	Total vigorous physical activity<br>Single-item question on total vigorous physical activity	No significant change in total vigorous physical activity (three or more times a week) in retirement (OR 1.06 [95% CI=0.89, 1.24])	Poor (1)
Parise (2004)[21]<br>U.S.	Cohort<br>(6 years)<br>Population-based	N=2091<br>54–99 years<br>40.5% male<br>76.2% retired at baseline<br>78.3% retired at follow-up	Leisure physical activity<br>Based on list of leisure activities	No significant change in leisure-time physical activity in retirement	Poor (0)
Zheng (2008)[22]<br>U.S.	Cohort<br>(6 years)<br>HRS<br>(Wave 3–6)<br>Population-based	N=3936<br>M 54.3 years (SD 2.0)<br>Male only<br>All employed at baseline<br>52.1% retired at follow-up	Total vigorous physical activity<br>Single-item question on total vigorous physical activity	Total vigorous physical activity (three or more times a week) decreased in retirement (OLS −0.049, $p<0.05$)	Modest (2)

Note: Studies are ordered by type (published articles followed by PhD theses) and then alphabetically by first author.

HRS, Health and Retirement Study; OLS, ordinary least-squares regression; RR, risk ratio

References for Appendix B

1. Berger U, Der G, Hannah MK, Mutrie N. The impact of retirement on physical activity. Ageing Soc 2005;25(2):181–95.
2. Brown WJ, Heesch KC, Miller YD. Life events and changing physical activity patterns in women at different life stages. Ann Behav Med 2009;37(3):294–305.
3. Brown WJ, Burton NW, Marshall AL, Miller YD. Reliability and validity of a modified self-administered version of the Active Australia physical activity survey in a sample of mid-age women. Aust NZ J Public Health 2008;32(6):535–41.
4. Chung S, Domino ME, Stearns SC, Popkin BM. Retirement and physical activity: analyses by occupation and wealth. Am J Prev Med 2009;36(5):422–8.
5. Evenson KR, Rosamond WD, Cai J, Diez-Roux AV, Brancati FL. Influence of retirement on leisure-time physical activity: The Atherosclerosis Risk in Communities Study. Am J Epidemiol 2002;155(8):692–9.

6. Baecke J, Burema J, Frijters J. A short questionnaire for the measurement of habitual physical activity in epidemiological studies. Am J Clin Nutr 1982;36:936–42.
7. Fonseca AM, Paúl C. Health and aging: Does retirement transition make any difference? Rev Clin Gerontol 2003;13(3):257–60.
8. Glamser FD, Hayslip B. The Impact of Retirement on Participation in Leisure Activities. Ther Recreation J 1985;19:28–38.
9. Henkens K, van Solinge H, Gallo WT. Effects of retirement voluntariness on changes in smoking, drinking and physical activity among Dutch older workers. Eur J Public Health 2008;18(6):644–9.
10. Mein GK, Shipley MJ, Hillsdon M, Ellison GT, Marmot MG. Work, retirement and physical activity: cross-sectional analyses from the Whitehall II study. Eur J Public Health 2005;15(3):317–22.
11. Midanik LT, Soghikian K, Ransom LJ, Tekawa IS. The effect of retirement on mental-health and health behaviors—the Kaiser Permanente Retirement Study. J Gerontol B Psychol Sci Soc Sci 1995;50(1):S59–S61.
12. Parnes H, Less L. Variation in selected forms of leisure activity among elderly males. In: Current perspectives on aging and the life cycle: Work, retirement and social policy. Greenwich CT: JAI Press, 1985.
13. Patrick JM, Bassey EJ, Irving JM, Blecher A, Fentem PH. Objective measurements of customary physical activity in elderly men and women before and after retirement. Q J Exp Physiol 1986;71(1):47–58.
14. Slingerland AS, van Lenthe FJ, Jukema J, et al. Aging, retirement, and changes in physical activity: prospective cohort findings from the GLOBE Study. Am J Epidemiol 2007;165(12):1356–63.
15. Touvier M, Bertrais S, Charreire H, Vergnaud AC, Hercberg S, Oppert JM. Changes in leisure-time physical activity and sedentary behaviour at retirement: a prospective study in middle-aged French subjects. Int J Behav Nutr Phys Act 2010;7:1–14.
16. Vuillemin A, Oppert JM, Guillemin F, et al. Self-administered questionnaire compared with interview to assess past-year physical activity. Med Sci Sports Exerc 2000;32:1119–24.
17. Wells YD, Kendig HL. Psychological resources and successful retirement. Aust Psychol 1999;34(2):111–5.
18. Wister A. The effects of socioeconomic status on exercise and smoking: age-related differences. J Aging Health 1996;8(4):467.
19. Chung S. Retirement and obesity among the near elderly [dissertation]. Chapel Hill: University of North Carolina, 2005.
20. Nekuda JD. The effect of retirement on vigorous physical activity controlling for the socio-demographic variables of age, gender, education, income, race and perceived health status affect [dissertation]. Lincoln NE: University of Nebraska, 2009.
21. Parise C. Characteristics of older adult exercisers [dissertation]. Davis CA: University of California, Davis, 2004.
22. Zheng Y. Economic determinants of obesity among old Americans [dissertation]. Santa Monica CA: Pardee RAND Graduate School, 2008.
